# Supplementary material for: Comparison of First- and Second-Generation Drug-Eluting Stents in Patients with ST-Segment Elevation Myocardial Infarction Based on Pre-Percutaneous Coronary Intervention Thrombolysis in Myocardial Infarction Flow Grade
Source: J Clin Med. 2021 Jan 19;10(2):367. doi: 10.3390/jcm10020367 (PMC7835978; doi:10.3390/jcm10020367)
Supplement: Supplementary file 1 [file jcm-10-00367-s001.pdf]

Supplementary Materials

Table S1. Baseline characteristics characteristic according to types of deployed stents in pre-TIMI 0/1 patients.

	**SES** (***n*** **= 2289)**	**PES** (***n*** **= 2029)**	**ZES** (***n*** **= 3336)**	**EES** (***n*** **= 3889)**	**BES** (***n*** **= 1062)**	***p*** **value**
Male, *n* (%)	1708 (74.6)	1491 (73.5)	2489 (74.6)	2992 (76.9)	846 (79.7)	<0.001
Age, years	61.8 ± 12.6	63.0 ± 12.7	62.6 ± 12.8	62.3 ± 12.6	61.7 ± 12.4	0.013
LVEF, %	50.0 ± 11.2	50.3 ± 11.4	50.9 ± 11.1	50.0 ± 10.6	51.1 ± 10.5	0.002
BMI, kg/m^2	24.0 ± 3.1	24.1 ± 3.1	24.3 ± 3.2	24.1 ± 3.2	24.1 ± 3.2	0.021
Killip classification						
I	1638 (71.6)	1527 (75.3)	2489 (74.6)	2989 (76.9)	874 (82.3)	<0.001
II	365 (15.9)	259 (12.8)	421 (12.6)	437 (11.2)	81 (7.6)	<0.001
III	123 (5.4)	102 (5.0)	185 (5.5)	215 (5.5)	32 (3.0)	0.016
IV	164 (7.2)	141 (6.9)	241 (7.2)	248 (6.4)	75 (7.1)	0.644
SBP, mmHg	124.9 ± 28.1	125.5 ± 28.2	126.1 ± 28.4	127.5 ± 29.0	127.4 ± 28.1	0.004
DBP, mmHg	77.5 ± 17.1	77.6 ± 17.0	77.7 ± 17.6	78.3 ± 17.7	78.3 ± 17.1	0.350
Cardiogenic shock, *n* (%)	164 (7.2)	141 (6.9)	241 (7.2)	248 (6.4)	75 (7.1)	0.644
CPR on admission, *n* (%)	47 (2.1)	38 (1.9)	158 (4.7)	258 (6.6)	93 (8.8)	<0.001
Hypertension, *n* (%)	1027 (44.9)	913 (45.0)	1498 (44.9)	1790 (46.0)	453 (42.7)	0.403
Diabetes mellitus, *n* (%)	596 (26.0)	472 (23.3)	805 (24.1)	936 (24.1)	248 (23.4)	0.229
Dyslipidemia, *n* (%)	191 (8.3)	168 (8.3)	351 (10.5)	433 (11.1)	108 (10.2)	<0.001
Previous MI, *n* (%)	56 (2.4)	53 (2.6)	88 (2.6)	137 (3.5)	25 (2.4)	0.048
Previous PCI, *n* (%)	82 (3.6)	61 (3.0)	152 (4.6)	183 (4.7)	41 (3.9)	0.010
Previous CABG, *n* (%)	8 (0.3)	7 (0.3)	8 (0.2)	14 (0.4)	3 (0.3)	0.906
Previous CVA, *n* (%)	140 (6.1)	97 (4.8)	165 (4.9)	177 (4.6)	54 (5.1)	0.097
Previous HF, *n* (%)	24 (1.0)	16 (0.8)	24 (0.7)	40 (1.0)	6 (0.6)	0.387
Current smokers, *n* (%)	1094 (47.8)	970 (47.8)	1573 (47.2)	1772 (45.6)	520 (49.0)	0.200
Peak CK-MB, mg/dL	216.0 ± 263.8	202.9 ± 301.8	203.6 ± 273.2	182.1 ± 211.2	184.3 ± 271.9	<0.001
Peak Troponin-I, ng/mL	58.9 ± 76.0	70.2 ± 140.4	82.9 ± 505.5	72.4 ± 127.0	81.1 ± 114.6	0.024
Blood glucose, mg/dL	176.8 ± 78.6	175.7 ± 81.6	177.7 ± 80.3	178.3 ± 78.5	173.7 ± 69.5	0.467
NT-ProBNP (pg/mL)	1830.4 ± 3858.1	1854.9 ± 3273.8	1712.4 ± 6820.8	1628.0 ± 3414.4	1424.3 ± 2882.5	0.062
High-sensitivity CRP (mg/dL)	10.5 ± 44.4	16.7 ± 69.8	9.8 ± 41.0	8.9 ± 37.2	6.7 ± 16.7	<0.001
Serum creatinine (mg/L)	1.152 ± 1.31	1.09 ± 0.89	1.11 ± 1.47	1.08 ± 1.02	0.98 ± 1.17	0.004
Total cholesterol, mg/dL	184.9 ± 44.0	184.7 ± 45.2	186.8 ± 45.1	183.4 ± 45.9	181.9 ± 41.5	0.006

Triglyceride, mg/L	123.4 ± 94.5	131.8 ± 126.1	137.7 ± 111.6	137.5 ± 110.4	134.3 ± 118.6	<0.001
HDL cholesterol, mg/L	45.2 ± 21.7	44.7 ± 17.9	44.0 ± 16.8	43.4 ± 13.8	42.3 ± 16.2	<0.001
LDL cholesterol, mg/L	117.7 ± 37.8	118.5 ± 39.0	117.8 ± 39.8	115.8 ± 38.7	115.9 ± 38.2	0.081
Discharge medications, *n* (%)						
Aspirin, *n* (%)	2220 (97.0)	1959 (96.6)	3235 (97.0)	3733 (96.0)	1026 (96.6)	0.001
Clopidogrel, *n* (%)	2231 (97.5)	1971 (97.1)	2975 (89.2)	3168 (81.5)	783 (73.7)	<0.001
Ticagrelor, *n* (%)	4 (0.2)	2 (0.1)	170 (5.1)	372 (9.6)	133 (12.5)	<0.001
Prasugrel, *n* (%)	6 (0.3)	0 (0.0)	99 (3.0)	197 (5.1)	106 (10.0)	<0.001
Cilostazole, *n* (%)	629 (27.5)	827 (40.8)	643 (19.3)	695 (17.9)	110 (10.4)	<0.001
Beta-blockers, *n* (%)	1570 (68.6)	1434 (70.7)	2549 (76.4)	3177 (81.7)	884 (83.2)	<0.001
ACEIs/ARBs, *n* (%)	1721 (75.2)	1560 (76.9)	2512 (75.3)	2997 (77.1)	823 (77.5)	0.208
CCBs, *n* (%)	157 (6.9)	109 (5.4)	93 (2.8)	120 (3.1)	30 (2.8)	<0.001
Lipid-lowering agents	2060 (90.0)	1825 (90.0)	2979 (89.3)	3583 (92.1)	939 (88.4)	0.185
IRA						
Left main, *n* (%)	21 (0.9)	20 (1.0)	33 (1.0)	42 (1.1)	7 (0.7)	0.804
LAD, *n* (%)	1229 (53.7)	915 (45.1)	1601 (48.0)	2017 (51.9)	534 (50.3)	<0.001
LCx, *n* (%)	233 (10.2)	217 (10.7)	293 (8.8)	350 (9.0)	105 (9.9)	0.095
RCA, *n* (%)	806 (35.2)	877 (43.2)	1409 (42.2)	1480 (38.1)	416 (39.2)	<0.001
Treated vessel						
Left main, *n* (%)	25 (1.1)	35 (1.7)	45 (1.3)	68 (1.7)	7 (0.7)	0.032
LAD, *n* (%)	1344 (58.7)	1055 (52.0)	1813 (54.3)	2283 (58.7)	611 (57.5)	<0.001
LCx, *n* (%)	358 (15.6)	340 (16.8)	482 (14.4)	609 (15.7)	158 (14.9)	0.225
RCA, *n* (%)	890 (38.9)	966 (47.6)	1544 (46.3)	1636 (42.1)	456 (42.9)	<0.001
Extent of CAD						
Single-vessel disease, *n* (%)	1089 (47.6)	966 (47.6)	1820 (54.6)	2076 (53.4)	696 (65.5)	<0.001
Two-vessel disease, *n* (%)	705 (30.8)	634 (31.2)	952 (28.5)	1162 (29.9)	251 (23.6)	<0.001
≥ Three-vessel disease, *n* (%)	495 (21.6)	429 (21.1)	564 (16.9)	651 (16.7)	115 (10.8)	<0.001
ACC/AHA lesion type						
Type B1, *n* (%)	326 (14.2)	276 (13.6)	434 (13.0)	497 (12.8)	115 (10.8)	0.082
Type B2, *n* (%)	474 (20.7)	447 (22.0)	877 (26.3)	1028 (26.4)	400 (37.3)	<0.001
Type C, *n* (%)	1188 (51.9)	1165 (57.4)	1718 (51.5)	2082 (53.5)	522 (49.2)	<0.001
In-hospital GP IIb/IIIa inhibitor	278 (12.1)	340 (16.8)	679 (20.4)	925 (23.8)	226 (21.3)	<0.001
IVUS, *n* (%)	149 (6.5)	118 (5.8)	531 (15.9)	689 (17.7)	164 (15.4)	<0.001
OCT, *n* (%)	0 (0.0)	0 (0.0)	4 (0.1)	13 (0.3)	4 (0.4)	0.002
FFR, *n* (%)	4 (0.2)	0 (0.0)	17 (0.5)	29 (0.7)	17 (1.6)	<0.001
Door-to-balloon time	59.5 (46.0-71.5)	60.0 (45.5-73.0)	60.0 (45.0-75.0)	59.5 (45.0-72.0)	60.0 (46.0-76.0)	0.288

Culprit-only PCI	810 (35.4)	634 (31.2)	1492 (44.7)	2231 (57.4)	716 (67.4)	<0.001
Multivessel PCI	1479 (64.6)	1395 (68.8)	1844 (55.3)	1658 (42.6)	346 (32.6)	<0.001
Completeness of PCI						
CR, *n* (%)	990 (66.9)	907 (65.0)	1272 (69.0)	1110 (67.0)	224 (64.7)	0.062
IR, *n* (%)	489 (33.1)	488 (34.5)	572 (31.0)	548 (33.0)	122 (35.3)	0.062
Stent diameter (mm)	3.115 ± 0.34	3.21 ± 0.42	3.20 ± 0.43	3.18 ± 0.43	3.19 ± 0.40	<0.001
Stent length (mm)	26.3 ± 6.3	26.4 ± 7.0	26.4 ± 9.6	27.6 ± 11.1	24.2 ± 8.0	<0.001
Number of stents	1.38 ± 0.69	1.46 ± 0.80	1.36 ± 0.68	1.38 ± 0.70	1.32 ± 0.65	<0.001

Values are means ± SD or numbers and percentages or as median (quartiles 1–3). The *p* values for continuous data obtained from the analysis of variance. The *p* values for categorical data from chi-square or Fisher's exact test.; SES, sirolimus-eluting stent; PES, paclitaxel-eluting stent; ZES, zotarolimus-eluting stent; EES, everolimus-eluting stent; BES, biolimus-eluting stent; LVEF, left ventricular ejection fraction; BMI, body mass index; SBP, systolic blood pressure; DBP, diastolic blood pressure; STEMI, ST-segment elevation myocardial infarction, NSTEMI, non-STEMI; CPR, cardiopulmonary resuscitation; MI, myocardial infarction; PCI, percutaneous coronary intervention; CABG, coronary artery bypass graft; HF, heart failure; CVA, cerebrovascular accidents; CK-MB, creatine kinase myocardial band; NT-ProBNP, N-terminal pro-brain natriuretic peptide; CRP, C-reactive protein; HDL, high-density lipoprotein; LDL, low-density lipoprotein; ACEIs, angiotensin converting enzyme inhibitors; ARBs, angiotensin receptor blockers; CCBs, calcium channel blockers; IRA, infarct-related artery; LAD, left anterior descending coronary artery; LCx, left circumflex coronary artery; RCA, right coronary artery; CAD, coronary artery disease; ACC/AHA, American College of Cardiology/American Heart Association; GP, glycoprotein; IVUS, intravascular ultrasound; OCT, optical coherence tomography; FFR, fractional flow reserve; CR, complete revascularization; IR, incomplete revascularization.

Table S2. Baseline characteristics characteristic according to types of deployed stents in pre-TIMI 2/3 patients.

	SES (*n* = 1141)	PES (*n* = 905)	ZES (*n* = 1116)	EES (*n* = 1331)	BES (*n* = 391)	*p* value
Male, *n* (%)	843 (74.6)	690 (73.5)	836 (74.6)	1027 (76.9)	298 (79.7)	0.386
Age, years	62.2 ± 12.6	63.2 ± 12.1	63.3 ± 13.1	63.3 ± 12.4	63.5 ± 13.2	0.142
LVEF, %	51.8 ± 11.9	51.9 ± 11.7	51.4 ± 11.6	51.5 ± 11.3	52.5 ± 10.3	0.547
BMI, kg/m^2	23.8 ± 3.1	23.7 ± 3.0	23.9 ± 3.2	23.9 ± 3.1	23.9 ± 3.4	0.667
Killip classification						
I	873 (76.5)	697 (77.0)	908 (81.4)	1077 (80.9)	322 (84.9)	<0.001
II	150 (13.1)	109 (12.0)	98 (8.8)	101 (7.6)	31 (7.9)	<0.001
III	58 (5.1)	60 (6.6)	55 (4.9)	86 (6.5)	14 (3.6)	0.083
IV	60 (5.3)	39 (4.3)	55 (4.9)	67 (5.0)	14 (3.6)	0.651
SBP, mmHg	128.4 ± 27.6	127.9 ± 26.8	129.0 ± 28.0	128.1 ± 28.0	128.8 ± 26.6	0.915
DBP, mmHg	78.5 ± 16.0	78.9 ± 16.5	78.5 ± 16.0	78.2 ± 16.3	79.5 ± 16.5	0.696
Cardiogenic shock, *n* (%)	60 (5.3)	39 (4.3)	55 (4.9)	67 (5.0)	14 (3.6)	0.651
CPR on admission, *n* (%)	33 (2.9)	24 (2.7)	49 (4.4)	85 (6.4)	24 (6.1)	<0.001
Hypertension, *n* (%)	520 (45.6)	414 (45.7)	522 (46.8)	614 (46.1)	197 (50.4)	0.547
Diabetes mellitus, *n* (%)	293 (25.7)	239 (26.4)	294 (26.3)	360 (27.0)	103 (26.3)	0.964
Dyslipidemia, *n* (%)	96 (8.4)	74 (8.2)	137 (12.3)	140 (10.5)	31 (7.9)	0.004
Previous MI, *n* (%)	25 (2.2)	17 (1.9)	38 (3.4)	36 (2.7)	12 (3.1)	0.214
Previous PCI, *n* (%)	42 (3.7)	24 (2.7)	47 (4.2)	77 (5.8)	14 (3.6)	0.005
Previous CABG, *n* (%)	1 (0.1)	4 (0.4)	2 (0.2)	3 (0.2)	1 (0.3)	0.562
Previous CVA, *n* (%)	60 (5.3)	42 (4.6)	80 (7.2)	65 (4.9)	24 (6.1)	0.073
Previous HF, *n* (%)	8 (0.7)	8 (0.9)	13 (1.2)	11 (0.8)	0 (0.0)	0.276
Current smokers, *n* (%)	558 (48.9)	429 (47.4)	500 (44.8)	617 (46.4)	175 (45.0)	0.339
Peak CK-MB, mg/dL	151.1 ± 29.52	122.8 ± 158.9	156.4 ± 266.9	135.7 ± 217.6	110.7 ± 125.1	0.001
Peak Troponin-I, ng/mL	48.4 ± 71.2	44.4 ± 65.0	47.3 ± 90.2	50.2 ± 102.8	48.0 ± 119.5	0.654
Blood glucose, mg/dL	173.0 ± 78.4	169.6 ± 80.0	171.1 ± 74.7	176.0 ± 79.8	174.0 ± 84.5	0.387
NT-ProBNP (pg/mL)	1656.5 ± 2801.7	2160.1 ± 4361.4	1830.1 ± 3969.6	1942.0 ± 4511.4	1731.0 ± 3588.1	0.054
High-sensitivity CRP (mg/dL)	9.6 ± 54.9	11.7 ± 89.1	9.1 ± 40.5	6.0 ± 12.7	6.0 ± 11.2	0.138
Serum creatinine (mg/L)	1.16 ± 1.91	1.17 ± 1.26	1.09 ± 1.00	1.05 ± 0.81	1.02 ± 0.89	0.071
Total cholesterol, mg/dL	184.5 ± 42.4	184.2 ± 43.4	182.2 ± 42.8	179.3 ± 43.7	181.7 ± 44.3	0.029
Triglyceride, mg/L	128.0 ± 110.4	121.2 ± 92.5	130.8 ± 106.9	132.8 ± 104.6	138.3 ± 131.5	0.057
HDL cholesterol, mg/L	46.8 ± 37.9	45.2 ± 13.9	44.2 ± 17.7	42.8 ± 14.0	41.7 ± 11.5	<0.001
LDL cholesterol, mg/L	119.3 ± 47.6	119.0 ± 39.4	113.3 ± 36.3	114.0 ± 48.8	113.0 ± 37.5	0.001

Discharge medications, *n* (%)						
Aspirin, *n* (%)	1121 (98.2)	879 (97.1)	1083 (97.0)	1278 (96.0)	379 (96.9)	0.487
Clopidogrel, *n* (%)	1110 (97.3)	888 (98.1)	994 (89.1)	1095 (82.3)	294 (75.2)	<0.001
Ticagrelor, *n* (%)	5 (0.4)	0 (0.0)	55 (4.9)	120 (9.0)	47 (12.0)	<0.001
Prasugrel, *n* (%)	2 (0.2)	0 (0.0)	35 (3.1)	70 (5.3)	38 (9.7)	<0.001
Cilostazole, *n* (%)	347 (30.4)	392 (43.3)	219 (19.6)	225 (16.9)	42 (10.7)	<0.001
Beta-blockers, *n* (%)	842 (73.8)	666 (73.6)	885 (79.3)	1087 (81.7)	331 (84.7)	<0.001
ACEIs/ARBs, *n* (%)	920 (80.6)	712 (78.7)	872 (78.1)	1054 (79.2)	313 (80.1)	0.644
CCBs, *n* (%)	97 (8.5)	65 (7.2)	55 (4.9)	77(5.8)	16 (4.1)	0.001
Lipid-lowering agents	1008 (88.3)	805 (89.0)	993 (89.0)	1171 (88.0)	342 (87.5)	0.425
IRA						
Left main, *n* (%)	23 (2.0)	23 (2.5)	30 (2.7)	44 (3.3)	7 (1.8)	0.266
LAD, *n* (%)	747 (65.5)	528 (58.3)	640 (57.3)	767 (57.6)	236 (60.4)	<0.001
LCx, *n* (%)	91 (8.0)	83 (9.2)	105 (9.4)	102 (7.7)	34 (8.7)	0.504
RCA, *n* (%)	280 (24.5)	271 (29.9)	341 (30.6)	418 (331.4)	114 (329.2)	0.003
Treated vessel						
Left main, *n* (%)	34 (3.0)	37 (4.1)	40 (3.6)	63 (4.7)	9 (2.3)	0.088
LAD, *n* (%)	798 (69.9)	601 (66.4)	723 (64.8)	875 (65.7)	263 (67.3)	0.096
LCx, *n* (%)	180 (15.8)	169 (18.7)	204 (18.3)	234 (17.6)	65 (16.6)	0.420
RCA, *n* (%)	341 (29.9)	340 (37.6)	420 (37.6)	492 (37.0)	135 (34.5)	<0.001
Extent of CAD						
Single-vessel disease, *n* (%)	543 (47.6)	413 (45.6)	540 (48.4)	669 (50.3)	235 (60.1)	<0.001
Two-vessel disease, *n* (%)	347 (30.4)	296 (32.7)	325 (29.1)	415 (31.2)	108 (27.6)	0.298
≥ Three-vessel disease, *n* (%)	251 (22.0)	196 (21.7)	251 (22.5)	247 (18.6)	48 (12.3)	<0.001
ACC/AHA lesion type						
Type B1, *n* (%)	274 (24.0)	170 (18.8)	192 (17.2)	217 (16.3)	87 (22.3)	<0.001
Type B2, *n* (%)	289 (25.3)	338 (37.3)	426 (38.2)	512 (38.5)	177 (45.3)	<0.001
Type C, *n* (%)	448 (39.3)	346 (38.2)	389 (34.9)	517 (38.8)	109 (27.9)	<0.001
In-hospital GP IIb/IIIa inhibitor	82 (7.2)	74 (8.2)	175 (15.7)	218 (16.4)	40 (10.2)	<0.001
IVUS, *n* (%)	87 (7.6)	57 (6.3)	219 (19.6)	315 (23.7)	71 (18.2)	<0.001
OCT, *n* (%)	0 (0.0)	0 (0.0)	5 (0.1)	4 (0.3)	3 (0.8)	0.022
FFR, *n* (%)	2 (0.2)	0 (0.0)	14 (1.3)	10 (0.8)	9 (2.3)	<0.001
Door-to-balloon time	60.5 (46.0-72.5)	60.5 (45.0-75.0)	59.5 (46.0-71.0)	60.0 (46.0-75.0)	59.5 (45.0-76.0)	0.118
Culprit-only PCI	371 (32.5)	265 (29.3)	465 (41.7)	691 (51.9)	251 (64.2)	<0.001
Multivessel PCI	770 (67.5)	640 (70.7)	651 (58.3)	640 (48.1)	140 (35.8)	<0.001
Completeness of PCI						

CR, *n* (%)	523 (67.9)	438 (68.4)	431 (66.2)	417 (65.2)	95 (67.9)	0.131
IR, *n* (%)	247 (32.1)	202 (31.6)	220 (33.8)	223 (34.8)	45 (32.1)	0.131
Stent diameter (mm)	3.12 ± 0.34	3.19 ± 0.41	3.18 ± 0.43	3.20 ± 0.44	3.18 ± 0.38	<0.001
Stent length (mm)	25.6 ± 6.7	25.2 ± 6.8	25.2 ± 9.5	26.4 ± 10.3	23.7 ± 9.9	<0.001
Number of stents	1.45 ± 0.73	1.57 ± 0.88	1.47 ± 0.76	1.43 ± 0.73	1.36 ± 0.67	<0.001

Values are means ± SD or numbers and percentages or as median (quartiles 1–3). The *p* values for continuous data obtained from the analysis of variance. The *p* values for categorical data from chi-square or Fisher's exact test.; SES, sirolimus-eluting stent; PES, paclitaxel-eluting stent; ZES, zotarolimus-eluting stent; EES, everolimus-eluting stent; BES, biolimus-eluting stent; LVEF, left ventricular ejection fraction; BMI, body mass index; SBP, systolic blood pressure; DBP, diastolic blood pressure; STEMI, ST-segment elevation myocardial infarction, NSTEMI, non-STEMI; CPR, cardiopulmonary resuscitation; MI, myocardial infarction; PCI, percutaneous coronary intervention; CABG, coronary artery bypass graft; HF, heart failure; CVA, cerebrovascular accidents; CK-MB, creatine kinase myocardial band; NT-ProBNP, N-terminal pro-brain natriuretic peptide; CRP, C-reactive protein; HDL, high-density lipoprotein; LDL, low-density lipoprotein; ACEIs, angiotensin converting enzyme inhibitors; ARBs, angiotensin receptor blockers; CCBs, calcium channel blockers; IRA, infarct-related artery; LAD, left anterior descending coronary artery; LCx, left circumflex coronary artery; RCA, right coronary artery; CAD, coronary artery disease; ACC/AHA, American College of Cardiology/American Heart Association; GP, glycoprotein; IVUS, intravascular ultrasound; OCT, optical coherence tomography; FFR, fractional flow reserve; CR, complete revascularization; IR, incomplete revascularization.

Table S3. Baseline characteristics characteristic according to types of deployed stents in the total study population.

	SES (*n* = 3430)	PES (*n* = 2934)	ZES (*n* = 4452)	EES (*n* = 5220)	BES (*n* = 1453)	*p* value
Male, *n* (%)	2551 (74.4)	2181 (74.3)	3225 (74.7)	4019 (77.0)	1144 (78.7)	<0.001
Age, years	61.9 ± 12.6	63.1 ± 12.7	62.8 ± 12.9	62.6 ± 12.6	62.2 ± 12.7	0.005
LVEF, %	50.6 ± 11.5	50.8 ± 11.5	51.0 ± 11.2	50.4 ± 10.8	51.5 ± 10.4	0.008
BMI, kg/m^2	23.9 ± 3.1	24.0 ± 3.1	24.2 ± 3.2	24.0 ± 3.2	24.1 ± 3.3	0.008
Killip classification						
I	2511 (73.2)	2224 (75.8)	3397 (76.3)	4066 (77.9)	1206 (83.0)	<0.001
II	515 (15.0)	368 (12.5)	519 (11.7)	538 (10.3)	112 (7.7)	<0.001
III	181 (5.43)	162 (5.5)	240 (5.4)	301 (5.8)	46 (3.2)	0.003
IV	224 (6.5)	180 (6.1)	296 (6.6)	315 (6.0)	89 (6.1)	0.726
SBP, mmHg	126.1 ± 28.0	126.3 ± 27.8	126.8 ± 28.3	127.7 ± 28.8	127.8 ± 27.7	0.051
DBP, mmHg	77.9 ± 16.7	78.0 ± 6.9	77.9 ± 17.2	78.3 ± 17.4	78.7 ± 17.0	0.520
Cardiogenic shock, *n* (%)	224 (6.5)	180 (6.1)	296 (6.6)	315 (6.0)	89 (6.1)	0.726
CPR on admission, *n* (%)	80 (2.3)	62 (2.1)	207 (4.6)	343 (6.6)	117 (8.1)	<0.001
Hypertension, *n* (%)	1547 (45.1)	1327 (45.2)	2020 (45.4)	2404 (46.1)	650 (44.7)	0.861
Diabetes mellitus, *n* (%)	889 (25.9)	711 (24.2)	1099 (24.7)	1296 (24.8)	351 (24.2)	0.536
Dyslipidemia, *n* (%)	287 (8.4)	242 (8.2)	488 (11.0)	573 (11.0)	139 (9.6)	<0.001
Previous MI, *n* (%)	81 (2.4)	70 (2.6)	126 (2.8)	173 (3.3)	37 (2.5)	0.045
Previous PCI, *n* (%)	124 (3.6)	85 (2.9)	199 (4.5)	260 (5.0)	55 (3.8)	<0.001
Previous CABG, *n* (%)	9 (0.3)	11 (0.4)	10 (0.2)	17 (0.3)	4 (0.3)	0.789
Previous CVA, *n* (%)	200 (5.8)	139 (4.7)	245 (5.5)	242 (4.6)	78 (5.4)	0.080
Previous HF, *n* (%)	32 (0.9)	24 (0.8)	37 (0.8)	51 (1.0)	6 (0.4)	0.334
Current smokers, *n* (%)	1652 (48.2)	1399 (47.7)	2073 (46.6)	2389 (45.8)	696 (47.9)	0.172
Peak CK-MB, mg/dL	194.6 ± 276.3	178.2 ± 268.6	191.8 ± 272.4	170.2 ± 213.8	164.5 ± 243.5	<0.001
Peak Troponin-I, ng/mL	55.4 ± 74.6	66.2 ± 122.8	74.0 ± 140.2	66.7 ± 121.7	72.2 ± 116.8	0.010
Blood glucose, mg/dL	175.6 ± 78.6	173.8 ± 81.0	176.0 ± 79.0	177.7 ± 78.9	173.8 ± 73.9	0.220
NT-ProBNP (pg/mL)	1772.5 ± 3542.4	1949.0 ± 3646.0	1741.9 ± 6229.7	1708.1 ± 3276.3	1506.8 ± 3090.0	0.027
High-sensitivity CRP (mg/dL)	10.2 ± 48.1	15.1 ± 51.1	9.7 ± 30.9	8.2 ± 32.7	6.5 ± 15.4	<0.001
Serum creatinine (mg/L)	1.15 ± 1.54	1.12 ± 1.02	1.11 ± 1.37	1.07 ± 0.97	0.99 ± 0.65	<0.001
Total cholesterol, mg/dL	184.8 ± 43.5	184.6 ± 44.6	185.7 ± 44.6	182.4 ± 45.4	181.8 ± 42.2	0.002
Triglyceride, mg/L	124.9 ± 100.1	128.5 ± 116.9	136.0 ± 110.5	136.3 ± 109.0	135.4 ± 122.2	<0.001
HDL cholesterol, mg/L	45.8 ± 28.2	44.8 ± 16.8	44.0± 17.0	43.3 ± 13.9	42.1 ± 15.1	<0.001
LDL cholesterol, mg/L	118.2 ± 41.4	118.6 ± 39.1	116.7 ± 39.0	115.4 ± 41.6	115.1 ± 38.0	0.001

Discharge medications, *n* (%)						
Aspirin, *n* (%)	3341 (97.4)	2838 (96.7)	4318 (97.0)	5011 (96.0)	1405 (96.7)	0.381
Clopidogrel, *n* (%)	3341 (97.4)	2859 (97.4)	3869 (89.2)	4263 (81.7)	1077 (74.1)	<0.001
Ticagrelor, *n* (%)	9 (0.3)	2 (0.1)	225 (5.1)	492 (9.4)	180 (12.4)	<0.001
Prasugrel, *n* (%)	8 (0.2)	0 (0.0)	134 (3.0)	267 (5.1)	144 (9.9)	<0.001
Cilostazole, *n* (%)	976 (28.5)	1219 (41.5)	862 (19.4)	920 (17.6)	152 (10.5)	<0.001
Beta-blockers, *n* (%)	2412 (70.3)	2100 (71.6)	3434 (77.1)	4264 (81.7)	1215 (83.6)	<0.001
ACEIs/ARBs, *n* (%)	2641 (77.0)	2272 (77.4)	3384 (76.0)	4051 (77.6)	1136 (78.2)	0.295
CCBs, *n* (%)	254 (7.4)	174 (5.9)	148 (3.3)	197 (3.8)	46 (3.2)	<0.001
Lipid-lowering agents	3068 (89.4)	2630 (89.6)	3972 (89.2)	4754 (91.1)	1281 (88.2)	0.102
IRA						
Left main, *n* (%)	44 (1.3)	43 (1.5)	63 (1.4)	86 (1.6)	14 (1.0)	0.336
LAD, *n* (%)	1976 (57.6)	1443 (49.2)	2241 (50.3)	2784 (53.3)	770 (53.0)	<0.001
LCx, *n* (%)	324 (9.4)	300 (10.2)	398 (8.9)	452 (8.7)	139 (9.6)	0.175
RCA, *n* (%)	1086 (31.7)	1148 (39.1)	1750 (39.3)	1898 (36.4)	530 (36.5)	<0.001
Treated vessel						
Left main, *n* (%)	59 (1.7)	72 (2.5)	85 (1.9)	131 (2.5)	16 (1.1)	0.002
LAD, *n* (%)	2142 (62.4)	1656 (56.4)	2536 (57.0)	3158 (60.5)	874 (60.2)	<0.001
LCx, *n* (%)	538 (15.7)	509 (17.3)	686 (15.4)	843 (16.1)	223 (15.3)	0.205
RCA, *n* (%)	1231 (35.9)	1306 (44.5)	1964 (44.1)	2128 (40.8)	591 (40.7)	<0.001
Extent of CAD						
Single-vessel disease, *n* (%)	1632 (47.6)	1379 (47.0)	2360 (53.0)	2745 (52.9)	931 (64.1)	<0.001
Two-vessel disease, *n* (%)	1052 (30.7)	930 (31.7)	1277 (28.7)	1577 (30.2)	359 (24.7)	<0.001
≥ Three-vessel disease, *n* (%)	746 (21.7)	625 (21.3)	815 (18.3)	898 (17.2)	163 (11.2)	<0.001
ACC/AHA lesion type						
Type B1, *n* (%)	600 (17.5)	446 (15.2)	626 (14.1)	714 (13.7)	202 (13.9)	<0.001
Type B2, *n* (%)	763 (22.2)	785 (26.8)	1303 (29.3)	1540 (29.5)	577 (39.7)	<0.001
Type C, *n* (%)	1636 (47.7)	1511 (51.5)	2017 (47.3)	2599 (49.8)	631 (43.4)	<0.001
In-hospital GP IIb/IIIa inhibitor	360 (10.5)	414 (14.1)	854 (19.2)	1143 (21.9)	266 (18.3)	<0.001
IVUS, *n* (%)	236 (6.9)	175 (6.0)	750 (15.8)	1004 (19.2)	235 (16.2)	<0.001
OCT, *n* (%)	0 (0.0)	0 (0.0)	9 (0.2)	17 (0.3)	7 (0.5)	<0.001
FFR, *n* (%)	6 (0.2)	0 (0.0)	31 (0.7)	39 (0.7)	26 (1.8)	<0.001
Door-to-balloon time	60.0 (46.0-72.5)	60.0 (45.0-75.0)	59.5 (45.0-75.0)	60.0 (45.0-75.0)	60.0 (45.0-76.0)	0.362
Culprit-only PCI	1181 (34.4)	899 (30.6)	1957 (44.0)	2922 (56.0)	967 (66.6)	<0.001
Multivessel PCI	2249 (65.6)	2035 (69.4)	2495 (56.0)	2298 (44.0)	486 (33.4)	<0.001
Completeness of PCI						

CR, *n* (%)	1954 (68.0)	2762 (69.5)	2762 (69.5)	2762 (69.5)	2762 (69.5)	0.180
IR, *n* (%)	920 (32.0)	1,213 (30.5)	1,213 (30.5)	1,213 (30.5)	1,213 (30.5)	0.180
Stent diameter (mm)	3.11 ± 0.34	3.20 ± 0.41	3.19 ± 0.43	3.19 ± 0.43	3.18 ± 0.41	<0.001
Stent length (mm)	26.1 ± 6.5	26.0 ± 7.0	26.1 ± 9.6	27.3 ± 10.9	24.1 ± 8.5	<0.001
Number of stents	1.40 ± 0.70	1.49 ± 0.82	1.39 ± 0.70	1.39 ± 0.71	1.33 ± 0.66	<0.001

Values are means ± SD or numbers and percentages or as median (quartiles 1–3). The *p* values for continuous data obtained from the analysis of variance. The *p* values for categorical data from chi-square or Fisher's exact test.; SES, sirolimus-eluting stent; PES, paclitaxel-eluting stent; ZES, zotarolimus-eluting stent; EES, everolimus-eluting stent; BES, biolimus-eluting stent; LVEF, left ventricular ejection fraction; BMI, body mass index; SBP, systolic blood pressure; DBP, diastolic blood pressure; STEMI, ST-segment elevation myocardial infarction, NSTEMI, non-STEMI; CPR, cardiopulmonary resuscitation; MI, myocardial infarction; PCI, percutaneous coronary intervention; CABG, coronary artery bypass graft; HF, heart failure; CVA, cerebrovascular accidents; CK-MB, creatine kinase myocardial band; NT-ProBNP, N-terminal pro-brain natriuretic peptide; CRP, C-reactive protein; HDL, high-density lipoprotein; LDL, low-density lipoprotein; ACEIs, angiotensin converting enzyme inhibitors; ARBs, angiotensin receptor blockers; CCBs, calcium channel blockers; IRA, infarct-related artery; LAD, left anterior descending coronary artery; LCx, left circumflex coronary artery; RCA, right coronary artery; CAD, coronary artery disease; ACC/AHA, American College of Cardiology/American Heart Association; GP, glycoprotein; IVUS, intravascular ultrasound; OCT, optical coherence tomography; FFR, fractional flow reserve; CR, complete revascularization; IR, incomplete revascularization.

Table S4. Independent predictors for MACEs and any revascularization in the total study population at 2 years.

	MACEs				Any repeat revascularization			
	Univariate		multivariate		Univariate		multivariate	
Variables	HR (95% CI)	*p* value	HR (95% CI)	*p* value	HR (95% CI)	*p* value	HR (95% CI)	*p* value
Group A1 vs. Group B1	1.201 (1.076 – 1.341)	0.001	1.212 (1.058 – 1.390)	0.006	1.832 (1.530 – 2.193)	<0.001	1.791 (1.449 – 2.214)	<0.001
Group A2 vs. Group B2	1.146 (0.954 – 1.375)	0.144	1.318 (1.054 – 1.649)	0.016	1.605 (1.218 – 2.115)	0.001	1.615 (1.164 – 2.240)	0.004
Male	1.507 (1.366 – 1.664)	<0.001	1.157 (1.022 – 1.309)	0.021	1.018 (0.853 – 1.214)	0.846	1.133 (0.919 – 1.397)	0.241
Age (≥ 65 years)	1.820 (1.657 – 1.999)	<0.001	1.197 (1.052 – 1.350)	0.003	1.166 (1.000 – 1.361)	0.051	1.369 (1.134 – 1.652)	0.001
LVEF (≤ 40%)	1.775 (1.594 – 1.977)	<0.001	1.235 (1.086 – 1.405)	0.001	1.173 (0.960 – 1.433)	0.118	1.006 (0.800 – 1.264)	0.961
Killip class III/IV	2.738 (2.460 – 3.047)	<0.001	1.416 (1.181 – 1.700)	<0.001	1.062 (0.834 – 1.352)	0.627	1.254 (0.887 – 1.771)	0.200
Cardiogenic shock	2.573 (2.248 – 2.944)	<0.001	1.275 (1.032 – 1.531)	<0.001	1.225 (0.854 – 1.757)	0.270	1.938 (1.142 – 3.289)	0.014
CPR on admission	4.651 (4.091 – 5.287)	<0.001	2.940 (2.498 – 3.461)	<0.001	1.715 (1.241 – 2.370)	0.001	2.008 (1.415 – 2.849)	<0.001
Hypertension	1.389 (1.266 – 1.523)	<0.001	1.120 (1.002 – 1.252)	0.051	1.140 (0.980 – 1.326)	0.088	1.051 (0.884 – 1.249)	0.572
Diabetes mellitus	1.519 (1.377 – 1.675)	<0.001	1.223 (1.089 – 1.374)	0.001	1.428 (1.214 – 1.679)	<0.001	1.328 (1.106 – 1.594)	0.002
Dyslipidemia	1.036 (0.886 – 1.212)	0.655	1.046 (0.878 – 1.246)	0.617	1.001 (0.777 – 1.288)	0.997	1.004 (0.769 – 1.310)	0.976
NT-ProBNP	1.002 (1.000 – 1.003)	<0.001	1.000 (0.999 – 1.001)	<0.001	1.000 (1.000 – 1.001)	0.583	1.000 (0.999 – 1.000)	0.931
Serum creatinine	1.051 (1.037 – 1.065)	<0.001	1.017 (0.985 – 1.050)	0.290	1.004 (0.947 – 1.065)	0.883	1.010 (0.945 – 1.080)	0.767
Total cholesterol	0.995 (0.994 – 0.996)	<0.001	0.998 (0.996 – 1.000)	0.050	1.001 (0.999 – 1.002)	0.526	1.000 (0.997 – 1.004)	0.826
Triglyceride	0.999 (0.998 – 0.999)	<0.001	1.000 (0.999 – 1.000)	0.423	1.000 (0.999 – 1.001)	0.918	1.000 (0.999 – 1.001)	0.422
HDL-cholesterol	0.986 (0.982 – 0.991)	<0.001	0.993 (0.988 – 0.997)	0.003	1.000 (0.995 – 1.004)	0.855	1.000 (0.996 – 1.004)	0.907
LDL-cholesterol	0.996 (0.995 – 0.997)	<0.001	1.002 (1.000 – 1.004)	0.690	1.001 (0.999 – 1.003)	0.361	1.001 (0.997 – 1.004)	0.726
Clopidogrel	1.418 (1.239 – 1.623)	<0.001	2.678 (2.147 – 3.341)	<0.001	1.475 (1.083 – 2.010)	0.014	1.233 (0.590 – 2.579)	0.578
Ticagrelor	1.824 (1.357 – 2.453)	<0.001	4.777 (3.263 – 6.993)	<0.001	1.309 (0.838 – 2.044)	0.236	1.158 (0.489 – 2.743)	0.739
Prasugrel	1.622 (1.155 – 2.278)	0.005	4.026 (2.644 – 6.130)	<0.001	1.505 (0.869 – 2.606)	0.144	1.232 (0.498 – 3.409)	0.652
Cilostazole	1.516 (1.344 – 1.711)	<0.001	1.268 (1.107 – 1.453)	0.001	1.222 (1.036 – 1.443)	0.018	1.086 (0.900 – 1.310)	0.388
Beta-blocker	3.382 (3.084 – 3.709)	<0.001	2.289 (2.047 – 2.559)	<0.001	1.325 (1.081 – 1.624)	0.007	1.322 (1.050 – 1.665)	0.017
LM (IRA)	3.448 (2.728 – 4.358)	<0.001	1.033 (0.842 – 1.662)	0.894	1.821 (1.073 – 3.092)	0.026	1.136 (0.488 – 2.615)	0.764
LM (treated vessel)	3.248 (2.657 – 3.970)	<0.001	2.365 (1.578 – 3.544)	<0.001	2.112 (1.405 – 3.174)	<0.001	1.994 (1.046 – 3.800)	0.036
Single-vessel disease	1.829 (1.663 – 2.012)	<0.001	1.221 (1.065 – 1.401)	0.004	2.271 (1.934 – 2.667)	<0.001	1.598 (1.281 – 1.994)	<0.001
≥ Three-vessel disease	1.913 (1.730 – 2.116)	<0.001	1.348 (1.177 – 1.544)	<0.001	2.290 (1.952 – 2.686)	<0.001	1.596 (1.303 – 1.954)	<0.001
ACC/AHA type B1	1.296 (1.126 – 1.493)	<0.001	1.151 (0.913 – 1.450)	0.233	1.021 (0.826 – 1.259)	0.849	1.068 (0.752 – 1.516)	0.713
ACC/AHA type B2/C	1.169 (1.044 – 1.309)	0.007	1.002 (0.830 – 1.208)	0.985	1.030 (0.862 – 1.230)	0.745	1.055 (0.781 – 1.427)	0.925
In hospital GP IIb/IIIa	1.138 (1.012 – 1.280)	0.031	1.152 (1.007 – 1.317)	0.039	1.078 (0.876 – 1.326)	0.479	1.012 (0.815 – 1.258)	0.911
IVUS	1.059 (0.922 – 1.216)	0.415	1.003 (0.858 – 1.172)	0.971	1.012 (0.810 – 1.264)	0.918	1.082 (0.847 – 1.381)	0.530
Culprit-only PCI	1.420 (1.294 – 1.557)	<0.001	1.231 (1.090 – 1.391)	0.001	1.485 (1.277 – 1.727)	<0.001	1.334 (1.100 – 1.618)	0.003
Stent diameter < 3.0mm	1.366 (1.233 – 1.514)	<0.001	1.120 (0.993 – 1.262)	0.057	1.162 (0.976 – 1.383)	0.092	1.075 (0.886 – 1.305)	0.462
Number of stent	1.153 (1.088 – 1.222)	<0.001	1.047 (0.968 – 1.132)	0.253	1.243 (1.136 – 1.359)	<0.001	1.051 (0.934 – 1.182)	0.413

HR, hazard ratio; CI, confidence interval; MACEs, major adverse cardiac events; LVEF, left ventricular ejection fraction; CPR, cardiopulmonary resuscitation; NT-ProBNP, N-terminal pro-brain natriuretic peptide; HDL, high density lipoprotein; LDL, low density lipoprotein; LM, left main coronary artery; IRA, infarct-related artery; ACC/AHA, American College of Cardiology/American Heart Association; GP, glycoprotein; IVUS, intravascular ultrasound; PCI, percutaneous coronary intervention.

Table S5. Baseline characteristics between pre-TIMI 0/1 and 2/3 in 1G-DES or 2G-DES groups. .

Variables	1G-DES (*n* = 6364)			2G-DES (*n* = 11527)		
	Pre-PCI TIMI 0/1 Group A 1G-DES (*n* = 4318)	Pre-PCI TIMI 2/3 Group C 1G-DES (*n* = 2046)	*p* value	Pre-PCI TIMI 0/1 Group B 2G-DES (*n* = 8544)	Pre-PCI TIMI 2/3 Group D 2G-DES (*n* = 2983)	*p* value
Male, *n* (%)	3199 (74.1)	1533 (74.9)	0.473	6534 (76.5)	2270 (76.1)	0.677
Age, years	62.4 ± 12.6	62.6 ± 12.4	0.493	62.3 ± 12.7	63.3 ± 12.8	<0.001
LVEF, %	50.2 ± 11.3	51.9 ± 11.8	<0.001	50.5 ± 10.8	51.7 ± 11.3	<0.001
BMI, kg/m^2	24.0 ± 3.1	23.8 ± 3.1	0.001	24.2 ± 3.2	23.9 ± 3.2	<0.001
Killip classification						
I	3165 (73.3)	1570 (76.7)	0.003	6554 (76.7)	2431 (81.5)	<0.001
II	624 (14.5)	259 (12.7)	0.053	969 (11.3)	246 (8.2)	<0.001
III	225 (5.2)	118 (5.8)	0.358	441 (5.2)	161 (5.4)	0.618
IV	305 (7.1)	99 (4.8)	0.001	580 (6.8)	145 (4.9)	<0.001
SBP, mmHg	125.2 ± 28.2	128.2 ± 27.2	<0.001	126.9 ± 28.6	128.4 ± 27.7	0.012
DBP, mmHg	77.5 ± 17.1	78.7 ± 16.2	0.012	78.0 ± 17.6	78.5 ± 16.2	0.222
Cardiogenic shock, *n* (%)	305 (7.1)	99 (4.8)	0.001	580 (6.8)	145 (4.9)	<0.001
CPR on admission, *n* (%)	85 (2.0)	57 (2.8)	0.045	525 (6.1)	159 (5.3)	0.116
Hypertension, *n* (%)	1940 (44.9)	934 (45.7)	0.589	3849 (45.0)	1398 (46.9)	0.086
Diabetes mellitus, *n* (%)	1068 (24.7)	532 (26.0)	0.276	2047 (24.0)	792 (26.6)	0.005
Dyslipidemia, *n* (%)	359 (8.3)	170 (8.3)	0.994	922 (10.8)	322 (10.8)	0.996
Previous MI, *n* (%)	109 (2.5)	42 (2.1)	0.290	259 (3.0)	88 (3.0)	0.848
Previous PCI, *n* (%)	143 (3.3)	66 (3.2)	0.881	387 (4.5)	142 (4.8)	0.604
Previous CABG, *n* (%)	15 (0.3)	5 (0.2)	0.634	27 (0.3)	6 (0.2)	0.428
Previous CVA, *n* (%)	237 (5.5)	102 (5.0)	0.437	415 (4.9)	176 (5.9)	0.029
Previous HF, *n* (%)	40 (0.9)	16 (0.8)	0.667	70 (0.8)	25 (0.8)	0.914
Current smokers, *n* (%)	2,064 (47.8)	987 (48.2)	0.742	4004 (46.9)	1364 (45.7)	0.284
Peak CK-MB, mg/dL	209.9 ± 282.3	138.8 ± 244.9	<0.001	191.0 ± 244.1	139.6 ± 225.6	<0.001
Peak Troponin-I, ng/mL	64.2 ± 111.1	46.6 ± 68.6	<0.001	77.1 ± 294.1	48.2 ± 98.7	<0.001
Blood glucose, mg/dL	176.3 ± 80.0	171.5 ± 79.0	0.025	177.4 ± 78.4	173.1 ± 79.0	0.013
NT-ProBNP (pg/mL)	1841.9 ± 3595.0	1879.2 ± 3584.3	0.698	1638.1 ± 4993.8	1884.9 ± 4338.3	0.010
Hs-sensitivity CRP (mg/dL)	13.4 ± 57.8	10.5 ± 79.3	0.138	9.1 ± 37.4	7.2 ± 26.9	0.003
Serum creatinine (mg/L)	1.12 ± 1.13	1.16 ± 1.66	0.313	1.08 ± 1.17	1.06 ± 0.90	0.445

Total cholesterol, mg/dL	184.9 ± 44.6	184.4 ± 42.8	0.731	184.7 ± 45.0	181.1 ± 44.1	<0.001
Triglyceride, mg/L	127.3 ± 110.5	125.0 ± 102.9	0.431	137.4 ± 112.0	133.6 ± 109.9	0.115
HDL cholesterol, mg/L	45.0 ± 20.0	46.1 ± 29.8	0.127	43.5 ± 15.8	43.2 ± 15.2	0.269
LDL cholesterol, mg/L	118.1 ± 38.4	119.1 ± 44.2	0.372	116.6 ± 39.1	113.5 ± 42.5	0.001
Discharge medications, *n* (%)						
Aspirin, *n* (%)	4192 (97.1)	1987 (97.1)	0.997	8237 (96.3)	2833 (95.0)	0.152
Clopidogrel, *n* (%)	4202 (97.3)	1998 (97.7)	0.423	7154 (83.7)	2514 (84.3)	0.485
Ticagrelor, *n* (%)	6 (0.1)	5 (0.2)	0.347	687 (8.0)	227 (7.6)	0.453
Prasugrel, *n* (%)	6 (0.1)	2 (0.1)	0.665	409 (4.8)	149 (5.0)	0.649
Cilostazole, *n* (%)	1,456 (33.7)	739 (36.1)	0.062	1,493 (17.5)	508 (17.0)	0.581
BBs, *n* (%)	3004 (69.6)	1508 (73.7)	0.001	6829 (79.4)	2429 (81.4)	0.076
ACEIs/ARBs, *n* (%)	3281 (76.0)	1632 (79.8)	0.001	6528 (76.4)	2353 (78.9)	0.006
CCBs, *n* (%)	266 (6.2)	162 (7.9)	0.009	250 (2.9)	149 (5.0)	<0.001
Lipid-lowering agents	3887 (90.0)	1811 (88.5)	0.215	7613 (89.0)	2639 (88.5)	0.717
IRA						
Left main, *n* (%)	41 (0.9)	46 (2.2)	<0.001	82 (1.0)	81 (2.7)	<0.001
LAD, *n* (%)	2144 (49.7)	1275 (62.3)	<0.001	4271 (50.0)	1726 (57.9)	<0.001
LCx, *n* (%)	450 (10.4)	174 (8.5)	0.017	773 (9.0)	252 (8.4)	0.322
RCA, *n* (%)	1683 (39.0)	551 (26.9)	<0.001	3418 (40.0)	924 (31.0)	<0.001
Treated vessel						
Left main, *n* (%)	60 (1.4)	71 (3.5)	<0.001	120 (1.4)	113 (3.8)	<0.001
LAD, *n* (%)	2399 (55.6)	1399 (68.4)	<0.001	4836 (56.6)	1951 (65.4)	<0.001
LCx, *n* (%)	698 (16.2)	349 (17.1)	0.370	1,283 (15.0)	527 (17.7)	0.001
RCA, *n* (%)	1856 (43.0)	681 (33.3)	<0.001	3757 (44.0)	1102 (36.9)	<0.001
Extent of CAD						
Single-vessel disease, *n* (%)	2055 (47.6)	956 (46.7)	0.518	4737 (55.4)	1523 (51.1)	<0.001
Two-vessel disease, *n* (%)	1339 (31.0)	643 (31.4)	0.737	2437 (28.5)	891 (29.9)	0.162
≥Three-vessel disease, *n* (%)	924 (21.4)	447 (21.8)	0.684	1370 (16.0)	569 (19.1)	<0.001
ACC/AHA lesion type						
Type B1, *n* (%)	602 (13.9)	444 (21.7)	<0.001	1075 (12.6)	521 (17.5)	<0.001
Type B2, *n* (%)	921 (21.3)	627 (30.6)	<0.001	2420 (28.3)	1188 (39.8)	<0.001
Type C, *n* (%)	2353 (54.5)	794 (38.8)	<0.001	4427 (51.8)	1051 (35.2)	<0.001
In-hospital GP IIb/IIIa inhibitor	1053 (24.4)	286 (14.0)	<0.001	1862 (21.8)	447 (15.0)	<0.001
IVUS, *n* (%)	267 (6.2)	144 (7.0)	0.195	1397 (16.4)	623 (20.9)	<0.001
OCT, *n* (%)	0 (0.0)	0 (0.0)	-	21 (0.2)	12 (0.4)	0.167
FFR, *n* (%)	4 (0.1)	2 (0.1)	0.950	65 (0.8)	34 (1.1)	0.062

Drug-eluting stents						
SES, *n* (%)	2289 (47.0)	1141 (55.8)	0.039			
PES, *n* (%)	2029 (53.0)	905 (44.2)	0.039			
ZES, *n* (%)				3336 (39.0)	1116 (37.4)	0.115
EES, *n* (%)				3889 (45.5)	1331 (44.6)	0.396
BES, *n* (%)				1062 (12.4)	391 (13.1)	0.337
Others, *n* (%)[a]				257 (3.0)	145 (4.9)	<0.001
Door-to-balloon time	59.5 (45.5-72.0)	60.0 (44.0-75.0)	0.585	60.0 (45.0-76.0)	60.5 (44.0-77.0)	0.569
Culprit-only PCI	1444 (33.4)	636 (31.1)	0.061	4569 (53.5)	1487 (49.8)	0.001
Multivessel PCI	2874 (66.6)	1410 (68.9)	0.061	3,975 (46.5)	1496 (50.2)	0.001
Completeness of PCI						
CR, *n* (%)	1954 (68.0)	988 (70.1)	0.153	2762 (69.5)	1069 (71.5)	0.147
IR, *n* (%)	920 (32.0)	422 (29.9)	0.153	1213 (30.5)	427 (28.5)	0.147
Stent diameter (mm)	3.15 ± 0.38	3.15 ± 0.37	0.896	3.19 ± 0.42	3.19 ± 0.42	0.821
Stent length (mm)	26.4 ± 6.7	25.4 ± 6.8	<0.001	26.6 ± 10.1	25.5 ± 9.9	<0.001
Number of stents	1.42 ± 0.74	1.50 ± 0.80	<0.001	1.36 ± 0.68	1.42 ± 0.73	<0.001

Values are means ± SD or numbers and percentages or as median (quartiles 1–3). The *p* values for continuous data were obtained from the unpaired t-test. The *p* values for categorical data from chi-square or Fisher's exact test. 1G-DES, first-generation drug-eluting stent; 2G, second-generation; Pre-PCI, pre-percutaneous coronary intervention; TIMI, Thrombolysis In Myocardial Infarction; LVEF, left ventricular ejection fraction; BMI, body mass index; SBP, systolic blood pressure; DBP, diastolic blood pressure; STEMI, ST-segment elevation myocardial infarction, NSTEMI, non-STEMI; CPR, cardiopulmonary resuscitation; MI, myocardial infarction; PCI, percutaneous coronary intervention; CABG, coronary artery bypass graft; HF, heart failure; CVA, cerebrovascular accidents; CK-MB, creatine kinase myocardial band; NT-ProBNP, N-terminal pro-brain natriuretic peptide; Hs-CRP, high sensitivity-C-reactive protein; HDL, high-density lipoprotein; LDL, low-density lipoprotein; ACEIs, angiotensin converting enzyme inhibitors; ARBs, angiotensin receptor blockers; CCBs, calcium channel blockers; IRA, infarct-related artery; LAD, left anterior descending coronary artery; LCx, left circumflex coronary artery; RCA, right coronary artery; CAD, coronary artery disease; ACC/AHA, American College of Cardiology/American Heart Association; GP, glycoprotein; IVUS, intravascular ultrasound; OCT, optical coherence tomography; FFR, fractional flow reserve; SES, sirolimus-eluting stent; PES, paclitaxel-eluting stent; ZES, zotarolimus-eluting stent; EES, everolimus-eluting stent; BES, biolimus-eluting stent; CR, complete revascularization; IR, incomplete revascularization.

Table S6. Baseline characteristics between pre-TIMI 0/1 and 2/3 groups or between 1G-DES and 2G-DES groups in the total study population.

Variables	Total population (n = 17891)					
	Pre-PCI TIMI 0/1 Group A1+B1 (n = 12862)	Pre-PCI TIMI 2/3 Group A2+B2 (n = 5029)	p value	1G-DES Group A1+A2 (n = 6364)	2G-DES Group B1+B2 (n = 11527)	p value
Male, n (%)	9733 (75.7)	3803 (75.6)	0.943	4732 (74.4)	8804 (76.4)	0.003
Age, years	62.3 ± 12.7	63.0 ± 12.6	0.001	62.5 ± 12.6	62.6 ± 12.7	0.648
LVEF, %	50.4 ± 11.0	51.8 ± 11.5	<0.001	50.7 ± 11.5	50.8 ± 10.9	0.723
BMI, kg/m^2	24.1 ± 3.2	23.8 ± 3.2	<0.001	23.9 ± 3.1	24.1 ± 3.2	0.007
Killip classification						
I	9719 (75.6)	4001 (79.6)	<0.001	4735 (74.4)	8985 (77.9)	<0.001
II	1593 (12.4)	505 (10.0)	<0.001	883 (13.9)	1215 (10.5)	<0.001
III	666 (5.2)	279 (5.5)	0.320	343 (5.4)	602 (5.2)	0.651
IV	885 (6.9)	244 (4.9)	<0.001	404 (6.3)	725 (6.3)	0.877
SBP, mmHg	126.3 ± 28.5	128.3 ± 27.5	<0.001	126.2 ± 27.9	127.3 ± 28.4	0.010
DBP, mmHg	77.9 ± 17.4	78.6 ± 16.2	0.014	77.9 ± 16.8	78.1 ± 17.2	0.394
Cardiogenic shock, n (%)	885 (6.9)	244 (4.9)	<0.001	404 (6.3)	725 (6.3)	0.874
CPR on admission, n (%)	610 (4.7)	216 (4.3)	0.200	142 (2.2)	684 (5.9)	<0.001
Hypertension, n (%)	5789 (45.0)	2332 (46.4)	0.100	2874 (45.2)	5247 (45.5)	0.644
Diabetes mellitus, n (%)	3115 (24.2)	1324 (26.3)	0.003	1600 (25.1)	2839 (24.6)	0.448
Dyslipidemia, n (%)	1281 (10.0)	492 (9.8)	0.723	529 (8.3)	1244 (10.8)	<0.001
Previous MI, n (%)	368 (2.9)	130 (2.6)	0.313	151 (2.4)	347 (3.0)	0.014
Previous PCI, n (%)	530 (4.1)	208 (4.1)	0.963	209 (3.3)	529 (4.6)	<0.001
Previous CABG, n (%)	42 (0.3)	11 (0.2)	0.285	20 (0.3)	33 (0.2)	0.780
Previous CVA, n (%)	652 (5.1)	278 (5.5)	0.214	339 (5.3)	591 (5.1)	0.565
Previous HF, n (%)	110 (0.9)	41(0.8)	0.851	56 (0.9)	95 (0.8)	0.696
Current smokers, n (%)	6068 (47.2)	2351 (46.7)	0.605	3051 (47.9)	5368 (46.6)	0.078
Peak CK-MB, mg/dL	197.3 ± 257.7	139.3 ± 233.6	<0.001	187.0 ± 272.9	177.7 ± 240.5	0.022
Peak Troponin-I, ng/mL	72.8 ± 196.4	47.5 ± 87.7	<0.001	58.5 ± 99.8	69.6 ± 121.7	<0.001
Blood glucose, mg/dL	177.0 ± 78.9	172.5 ± 79.0	0.001	174.7 ± 79.7	176.3 ± 78.6	0.220
NT-ProBNP (pg/mL)	1706.5 ± 4573.1	1882.6 ± 4048.1	0.012	1853.9 ± 3591.3	1702.0 ± 4833.8	0.017
Hs-sensitivity CRP (mg/dL)	10.5 ± 45.3	8.5 ± 54.6	0.021	12.5 ± 65.5	8.6 ± 35.0	<0.001
Serum creatinine (mg/L)	1.09 ± 1.16	1.10 ± 1.27	0.611	1.14 ± 1.32	1.07 ± 1.10	0.001
Total cholesterol, mg/dL	184.7 ± 44.9	182.5 ± 43.6	0.003	184.7 ± 44.0	183.8 ± 44.8	0.196

Triglyceride, mg/L	134.0 ± 111.6	130.1 ± 107.2	0.035	126.6 ± 108.1	136.4 ± 111.5	<0.001
HDL cholesterol, mg/L	44.0 ± 17.3	44.4 ± 22.4	0.336	45.3 ± 23.7	43.4 ± 15.6	<0.001
LDL cholesterol, mg/L	117.1 ± 38.8	115.8 ± 43.3	0.073	118.4 ± 40.4	115.8 ± 40.0	<0.001
Discharge medications, *n* (%)						
Aspirin, *n* (%)	12429 (96.6)	4820 (95.8)	0.307	6179 (97.1)	11070 (96.0)	0.152
Clopidogrel, *n* (%)	11356 (88.3)	4512 (89.7)	0.007	6200 (97.4)	9668 (83.9)	<0.001
Ticagrelor, *n* (%)	693 (5.4)	232 (4.6)	0.036	11 (0.2)	914 (7.9)	<0.001
Prasugrel, *n* (%)	415 (3.2)	151 (3.0)	0.442	8 (0.1)	558 (4.8)	<0.001
Cilostazole, *n* (%)	2949 (33.7)	1247 (24.8)	0.008	2195 (34.5)	2001 (17.4)	<0.001
BBs, *n* (%)	9833 (76.5)	3937 (78.3)	0.009	4512 (70.9)	9258 (80.3)	<0.001
ACEIs/ARBs, *n* (%)	9,809 (76.3)	3,985 (79.2)	<0.001	4913 (77.2)	8881 (77.0)	0.823
CCBs, *n* (%)	516 (4.0)	311 (6.2)	<0.001	428 (6.7)	399 (3.5)	<0.001
Lipid-lowering agents	11500 (89.4)	4450 (88.5)	0.287	5698 (89.5)	10252 (88.9)	0.304
IRA						
Left main, *n* (%)	123 (1.0)	127 (2.5)	<0.001	87 (1.4)	163 (1.4)	0.840
LAD, *n* (%)	6415 (49.9)	3001 (59.7)	<0.001	3419 (53.7)	5997 (52.0)	0.029
LCx, *n* (%)	1223 (9.5)	426 (8.5)	0.032	624 (9.8)	1025 (8.9)	0.043
RCA, *n* (%)	5101 (39.7)	1475 (29.3)	<0.001	2234 (35.1)	4342 (37.7)	0.001
Treated vessel						
Left main, *n* (%)	180 (1.4)	184 (3.7)	<0.001	131 (2.1)	233 (2.0)	0.866
LAD, *n* (%)	7235 (56.3)	3350 (66.6)	<0.001	3798 (59.7)	6787 (58.9)	0.297
LCx, *n* (%)	1981 (15.4)	876 (17.4)	0.001	1047 (16.5)	1810 (15.7)	0.190
RCA, *n* (%)	5613 (43.6)	1783 (35.5)	<0.001	2537 (39.9)	4859 (42.2)	0.003
Extent of CAD						
Single-vessel disease, *n* (%)	6792 (52.8)	2479 (49.3)	< 0.001	3011 (47.3)	6260 (54.3)	<0.001
Two-vessel disease, *n* (%)	3776 (29.4)	1534 (30.5)	0.132	1982 (31.1)	3328 (28.9)	0.001
≥Three-vessel disease, *n* (%)	2294 (17.8)	1016 (20.2)	<0.001	1371 (21.5)	1939 (16.8)	<0.001
ACC/AHA lesion type						
Type B1, *n* (%)	1677 (13.0)	965 (19,2)	<0.001	1046 (16.4)	1596 (13.8)	<0.001
Type B2, *n* (%)	3341 (26.0)	1815 (36.1)	<0.001	1548 (24.3)	3608 (31.3)	<0.001
Type C, *n* (%)	6780 (52.7)	1845 (36.7)	<0.001	3147 (49.5)	5478 (47.5)	0.014
In-hospital GP IIb/IIIa inhibitor	2480 (19.3)	603 (11.2)	<0.001	774 (12.2)	2309 (20.0)	<0.001
IVUS, *n* (%)	1664 (12.9)	767 (15.3)	<0.001	411 (6.5)	2020 (17.5)	<0.001
OCT, *n* (%)	21 (0.2)	12 (0.2)	0.332	0 (0.0)	33 (0.3)	<0.001
FFR, *n* (%)	69 (0.5)	36 (0.7)	0.157	6 (0.1)	99 (0.9)	<0.001
Drug-eluting stents						

SES, *n* (%)	2289 (17.8)	1141 (22.7)	<0.001	3430 (53.9)		
PES, *n* (%)	2029 (15.8)	905 (18.0)	<0.001	2934 (46.1)		
ZES, *n* (%)	3338 (26.0)	1117 (22.2)	<0.001		4452 (38.6)	
EES, *n* (%)	3893 (30.3)	1332 (26.5)	<0.001		5220 (45.3)	
BES, *n* (%)	1063 (8.3)	391 (7.8)	0.287		1453 (12.6)	
Others, *n* (%)[a]	257 (2.0)	145 (2.9)	<0.001		402 (3.5)	
Door-to-balloon time	60.0 (45.0-76.0)	60.5 (44.0-77.0)	0.585	60.0 (44.0-75.0)	60.0 (44.0-77.0)	0784
Culprit-only PCI	6013 (46.8)	2123 (42.2)	<0.001	2080 (32.7)	6056 (52.5)	<0.001
Multivessel PCI	6849 (53.2)	2906 (57.8)	<0.001	4284 (67.3)	5471 (47.5)	<0.001
Completeness of PCI						
CR, *n* (%)	4716 (68.9)	2057 (70.8)	0.009	2942 (68.7)	3831 (70.0)	0.274
IR, *n* (%)	2133 (31.1)	849 (29.2)	0.009	1342 (31.3)	1640 (30.0)	0.274
Stent diameter (mm)	3.18 ± 0.40	3.17 ± 0.37	0.618	3.15 ± 0.38	3.19 ± 0.42	<0.001
Stent length (mm)	26.5 ± 9.1	25.5 ± 8.7	<0.001	26.1 ± 6.7	26.3 ± 10.1	0.062
Number of stents	1.38 ± 0.70	1.46 ± 0.76	<0.001	1.44 ± 0.76	1.38 ± 0.69	<0.001

Values are means ± SD or numbers and percentages or as median (quartiles 1–3). The *p* values for continuous data were obtained from the unpaired t-test. The *p* values for categorical data from chi-square or Fisher's exact test. 1G-DES, first-generation drug-eluting stent; 2G, second-generation; Pre-PCI, pre-percutaneous coronary intervention; TIMI, Thrombolysis In Myocardial Infarction; LVEF, left ventricular ejection fraction; BMI, body mass index; SBP, systolic blood pressure; DBP, diastolic blood pressure; STEMI, ST-segment elevation myocardial infarction, NSTEMI, non-STEMI; CPR, cardiopulmonary resuscitation; MI, myocardial infarction; PCI, percutaneous coronary intervention; CABG, coronary artery bypass graft; HF, heart failure; CVA, cerebrovascular accidents; CK-MB, creatine kinase myocardial band; NT-ProBNP, N-terminal pro-brain natriuretic peptide; Hs-CRP, high sensitivity-C-reactive protein; HDL, high-density lipoprotein; LDL, low-density lipoprotein; ACEIs, angiotensin converting enzyme inhibitors; ARBs, angiotensin receptor blockers; CCBs, calcium channel blockers; IRA, infarct-related artery; LAD, left anterior descending coronary artery; LCx, left circumflex coronary artery; RCA, right coronary artery; CAD, coronary artery disease; ACC/AHA, American College of Cardiology/American Heart Association; GP, glycoprotein; IVUS, intravascular ultrasound; OCT, optical coherence tomography; FFR, fractional flow reserve; SES, sirolimus-eluting stent; PES, paclitaxel-eluting stent; ZES, zotarolimus-eluting stent; EES, everolimus-eluting stent; BES, biolimus-eluting stent; CR, complete revascularization; IR, incomplete revascularization.

A

Pre-PCI TIMI flow grade 0/1

Subgroup	1G-DES	2G-DES	HR (95% CI)	*p* value	*p*-for-interaction
All patients	517/4318	826/8544	1.20 (1.08-1.34)	0.001	
Ages, years					<0.001
≥ 65	291/1939	497/3701	1.08 (0.94-1.25)	0.281	
< 65	226/2379	329/4843	1.35 (1.14-1.60)	<0.001	
Sex					0.633
Male	351/3199	544/6534	1.28 (1.17-1.46)	<0.001	
Female	166/1119	282/2010	1.03 (0.85-1.25)	0.742	
LVEF, %					<0.001
≥ 40	382/3541	629/7253	1.20 (1.06-1.37)	0.004	
< 40	135/777	197/1291	1.11 (0.90-1.38)	0.347	
Hypertension					<0.001
Yes	266/1940	455/3849	1.12 (0.97-1.31)	0.129	
No	251/2378	371/4695	1.30 (1.10-1.52)	0.002	
Diabetes mellitus					<0.001
Yes	151/1068	275/2047	1.01 (0.83-1.23)	0.914	
No	366/3250	551/6497	1.29 (1.13-1.47)	<0.001	
Multivessel disease					<0.001
Yes	346/2263	488/3807	1.17 (1.02-1.34)	0.030	
No	171/2055	338/4737	1.12 (0.93-1.35)	0.230	
Cardiogenic shock					0.280
Yes	68/305	128/580	0.99 (0.74-1.33)	0.947	
No	449/4013	698/7964	1.24 (1.10-1.39)	<0.001	
CPR on admission					0.389
Yes	28/85	181/525	0.98 (0.66-1.46)	0.925	
No	489/4233	645/8019	1.40 (1.25-1.58)	<0.001	
In hospital GP IIb/IIIa					0.002
Yes	90/618	187/1862	1.42 (1.11-1.83)	0.006	
No	427/3700	639/6682	1.17 (1.03-1.32)	0.013	
Culprit-only PCI					0.425
Yes	247/1444	486/4569	1.56 (1.34-1.82)	<0.001	
No	270/2874	340/3975	1.08 (0.92-1.27)	0.339	
Stent diameter, mm					0.005
≥ 3.0	380/3358	567/6550	1.27 (1.12-1.45)	<0.001	
< 3.0	137/960	259/1994	1.06 (0.86-1.30)	0.588	

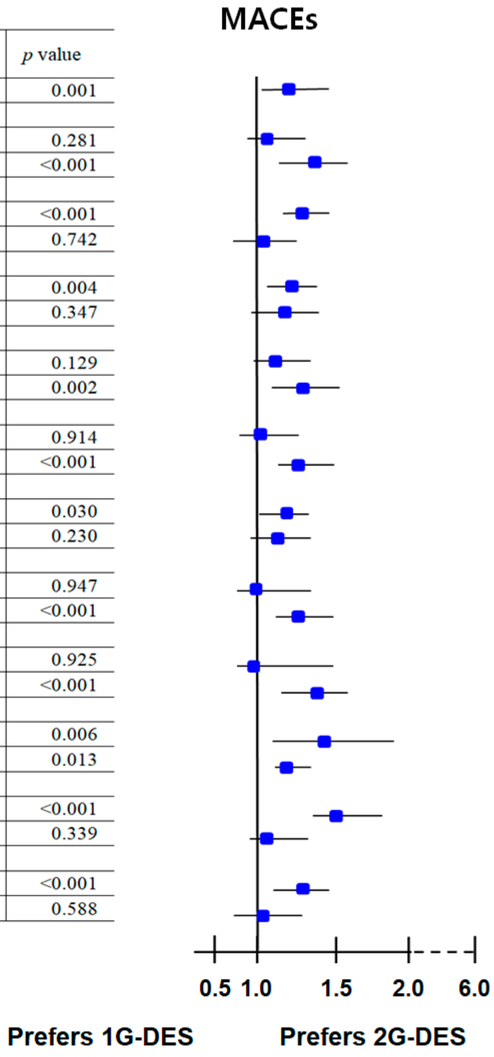

B

Pre-PCI TIMI flow grade 2/3

Subgroup	1G-DES	2G-DES	HR (95% CI)	*p* value	*p*-for-interaction
All patients	209/2046	257/2983	1.14 (0.95-1.38)	0.144	
Ages, years					0.033
≥ 65	110/925	153/1379	1.04 (0.81-1.32)	0.714	
< 65	99/1121	104/1604	1.31 (1.00-1.73)	0.052	
Sex					0.574
Male	151/1533	183/2270	1.18 (0.95-1.46)	0.135	
Female	58/513	74/713	1.06 (0.75-1.50)	0.729	
LVEF, %					0.204
≥ 40	165/1730	197/2573	1.21 (0.98-1.48)	0.083	
< 40	44/316	60/410	0.92 (0.63-1.36)	0.688	
Hypertension					0.039
Yes	105/934	135/1398	1.13 (0.87-1.46)	0.350	
No	104/1112	122/1585	1.17 (0.90-1.52)	0.241	
Diabetes mellitus					<0.001
Yes	75/532	95/792	1.15 (0.85-1.56)	0.353	
No	134/1514	162/2191	1.15 (0.92-1.45)	0.230	
Multivessel disease					<0.001
Yes	136/1090	161/1460	1.10 (0.88-1.38)	0.416	
No	73/956	96/1523	1.16 (0.86-1.58)	0.335	
Cardiogenic shock					<0.001
Yes	21/99	27/145	1.12 (0.64-1.99)	0.690	
No	188/1947	230/2838	1.15 (0.95-1.39)	0.159	
CPR on admission					0.934
Yes	19/57	48/159	1.11 (0.66-1.90)	0.689	
No	190/1989	209/2824	1.25 (1.03-1.52)	0.027	
In hospital GP IIb/IIIa					0.042
Yes	22/156	44/447	1.42 (0.85-2.36)	0.184	
No	187/1890	213/2536	1.14 (0.93-1.38)	0.202	
Culprit-only PCI					0.328
Yes	93/636	129/1487	1.63 (1.25-2.13)	<0.001	
No	116/1410	128/1496	0.94 (0.73-1.21)	0.634	
Stent diameter, mm					0.208
≥ 3.0	172/1626	181/2291	1.31 (1.06-1.61)	0.012	
< 3.0	37/420	76/692	0.75 (0.51-1.11)	0.148	

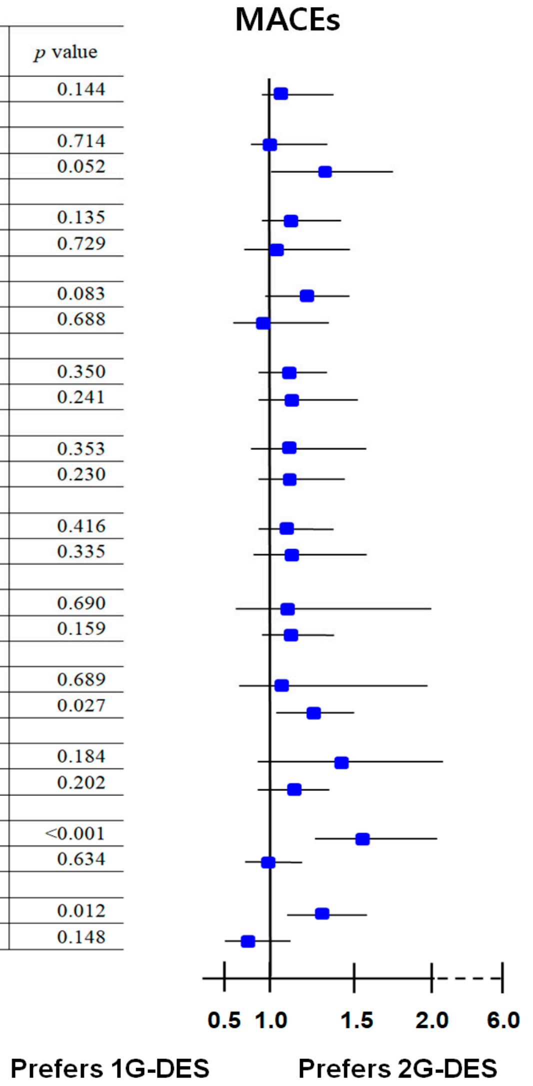

C

Pre-PCI TIMI flow grade 0/1

Subgroup	1G-DES	2G-DES	HR (95% CI)	*p* value	*p*-for-interaction
All patients	234/4318	240/8544	1.83 (1.53-2.19)	<0.001	
Ages, years					<0.001
≥ 65	91/1939	98/3701	1.66 (1.25-2.21)	0.001	
< 65	143/2379	142/4843	1.96 (1.56-2.48)	<0.001	
Sex					0.001
Male	179/3199	178/6534	1.95 (1.59-2.40)	<0.001	
Female	55/1119	62/2010	1.52 (1.05-2.18)	0.025	
LVEF, %					0.038
≥ 40	183/3541	205/7253	1.73 (1.42-2.12)	<0.001	
< 40	51/777	35/1291	2.30 (1.50-3.54)	<0.001	
Hypertension					<0.001
Yes	107/1940	125/3849	1.68 (1.24-2.08)	<0.001	
No	127/2378	115/4695	2.07 (1.61-2.67)	<0.001	
Diabetes mellitus					<0.001
Yes	68/1068	68/2047	1.80 (1.29-2.52)	0.001	
No	166/3250	172/6497	1.84 (1.49-2.28)	<0.001	
Multivessel disease					<0.001
Yes	168/2263	149/3807	1.83 (1.47-2.28)	<0.001	
No	66/2055	91/4737	1.55 (1.13-2.12)	0.007	
Cardiogenic shock					0.452
Yes	10/305	14/580	1.28 (0.57-2.89)	0.546	
No	224/4013	226/7964	1.87 (1.55-2.25)	<0.001	
CPR on admission					0.621
Yes	2/85	24/525	0.48 (0.11-2.03)	0.317	
No	232/4233	216/8019	1.96 (1.63-2.35)	<0.001	
In hospital GP IIb/IIIa					0.002
Yes	44/618	47/1862	2.72 (1.81-4.11)	<0.001	
No	190/3700	193/6682	1.68 (1.38-2.06)	<0.001	
Culprit-only PCI					0.001
Yes	107/1444	154/4569	2.08 (1.63-2.66)	<0.001	
No	127/2874	86/3975	2.00 (1.52-2.63)	<0.001	
Stent diameter, mm					0.003
≥ 3.0	181/3358	172/6550	1.96 (1.59-2.42)	<0.001	
< 3.0	53/960	68/1994	1.51 (1.05-2.16)	0.025	

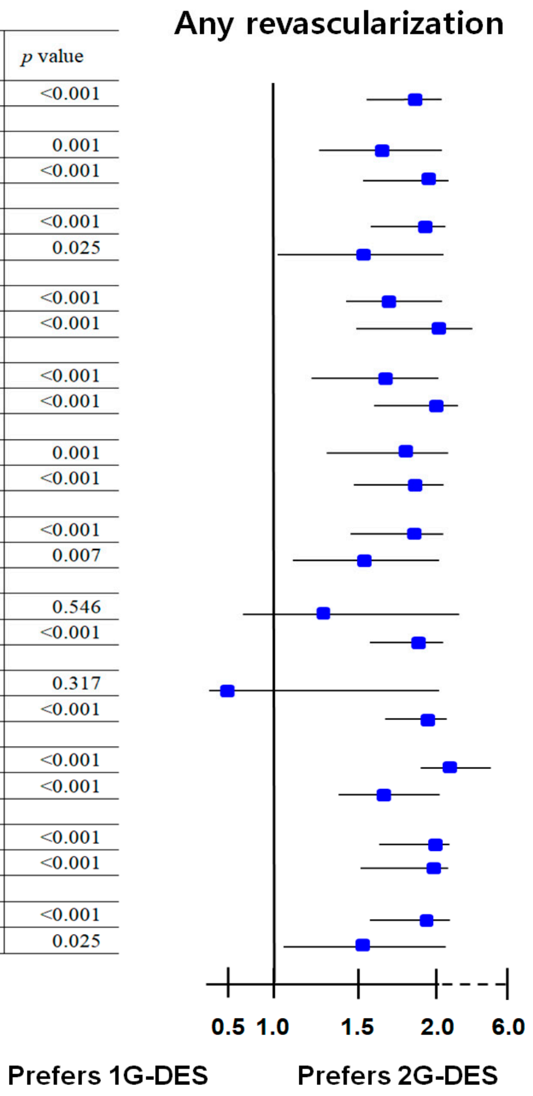

D

Pre-PCI TIMI flow grade 2/3

Subgroup	1G-DES	2G-DES	HR (95% CI)	*p* value	*p*-for-interaction
All patients	109/2046	94/2983	1.61 (1.22-2.12)	0.001	
Ages, years					0.005
≥ 65	40/925	38/1379	1.49 (0.96-2.32)	0.079	
< 65	69/1121	56/1604	1.68 (1.18-2.39)	0.004	
Sex					0.003
Male	84/1533	73/2270	1.62 (1.18-2.21)	0.003	
Female	25/513	21/713	1.58 (0.89-2.83)	0.122	
LVEF, %					<0.001
≥ 40	93/1730	80/2573	1.64 (1.22-2.21)	0.001	
< 40	16/316	14/410	1.41 (0.69-2.89)	0.247	
Hypertension					0.039
Yes	50/934	43/1398	1.66 (1.11-2.50)	0.015	
No	59/1112	51/1585	1.56 (1.07-2.27)	0.021	
Diabetes mellitus					0.002
Yes	35/532	42/792	1.20 (0.77-1.89)	0.419	
No	74/1514	52/2191	1.94 (1.36-2.77)	<0.001	
Multivessel disease					0.001
Yes	76/1090	63/1460	1.54 (1.10-2.15)	0.011	
No	33/956	31/1523	1.60 (0.98-2.61)	0.061	
Cardiogenic shock					0.722
Yes	3/99	4/145	1.09 (0.24-4.87)	0.909	
No	106/1947	90/2838	1.63 (1.23-2.16)	0.001	
CPR on admission					0.322
Yes	5/57	8/159	1.73 (0.57-5.30)	0.335	
No	104/1989	86/2824	1.64 (1.23-2.18)	0.001	
In hospital GP IIb/IIIa					0.032
Yes	11/156	12/447	2.54 (1.12-5.75)	0.026	
No	98/1890	82/2536	1.52 (1.13-2.04)	0.005	
Culprit-only PCI					<0.001
Yes	45/636	53/1487	1.89 (1.27-2.81)	0.002	
No	64/1410	41/1496	1.60 (1.08-2.37)	0.018	
Stent diameter, mm					0.068
≥ 3.0	90/1626	66/2291	1.85 (1.35-2.55)	<0.001	
< 3.0	19/420	28/692	1.00 (0.56-1.80)	0.993	

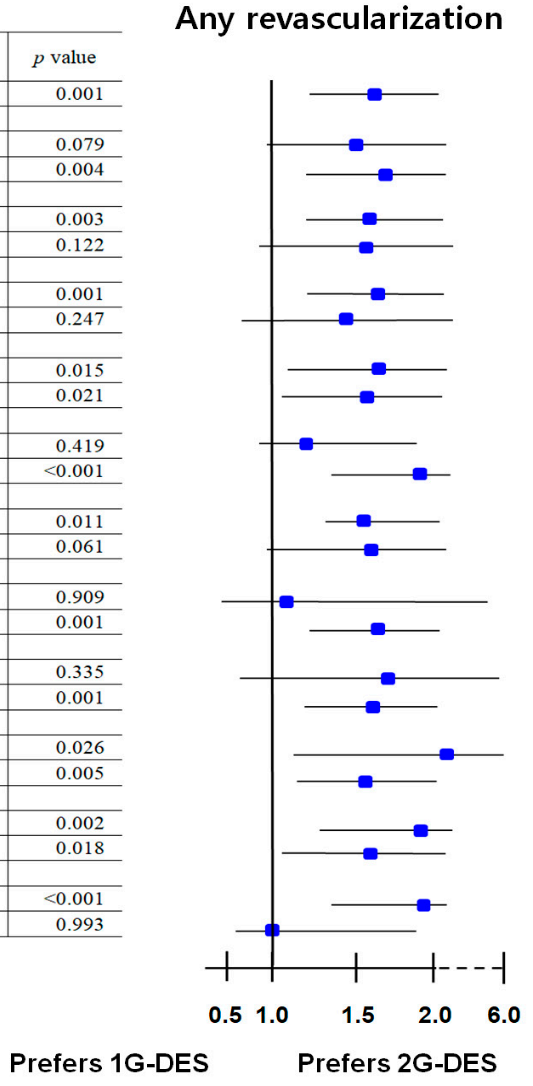

Figure S1. Subgroup analysis for MACEs (A, B) and any repeat revascularization (C, D) in pre-TIMI flow grade 0/1 (A, C) and 2/3 (B, D) groups.

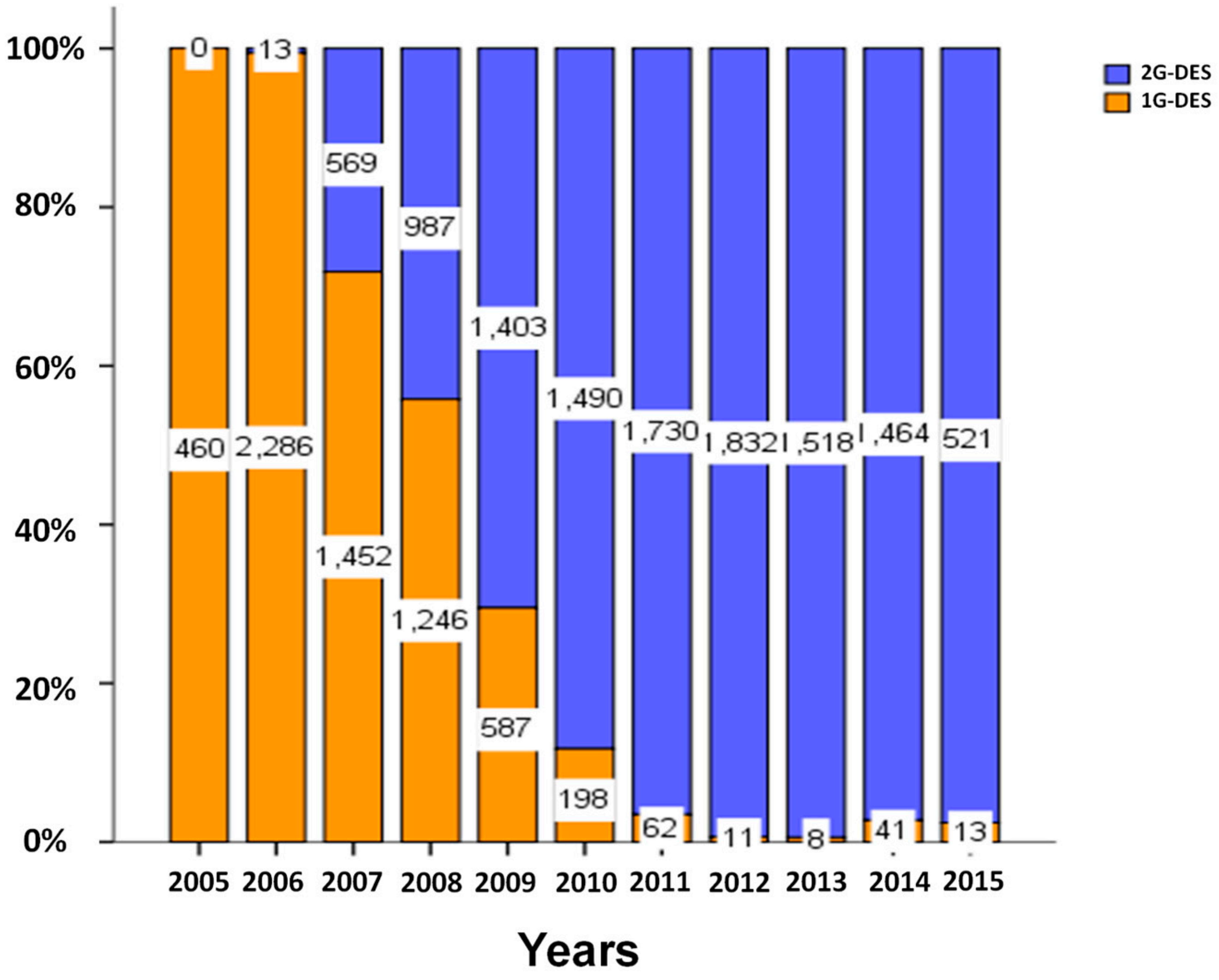

Figure S2. Temporal trends of 1G-DES and 2G-DES.
